# Magnetic Fingerprints in an All-Organic Radical Molecular Break Junction

**DOI:** 10.1021/acs.nanolett.2c02326

**Published:** 2022-10-07

**Authors:** Thomas Y. Baum, Saleta Fernández, Diego Peña, Herre S. J. van der Zant

**Affiliations:** † Kavli Institute of Nanoscience, Delft University of Technology, Lorentzweg 1, 2628 CJDelft, The Netherlands; ‡ Centro Singular de Investigación en Química Biolóxica e Materiais Moleculares (CiQUS) and Departamento de Química Orgánica, Universidade de Santiago de Compostela, Santiago de Compostela, Spain15782

**Keywords:** molecular electronics, organic radical, mechanically controlled break junction, Kondo effect, inelastic spin-flip spectroscopy, open-shell polycyclic aromatic hydrocarbons

## Abstract

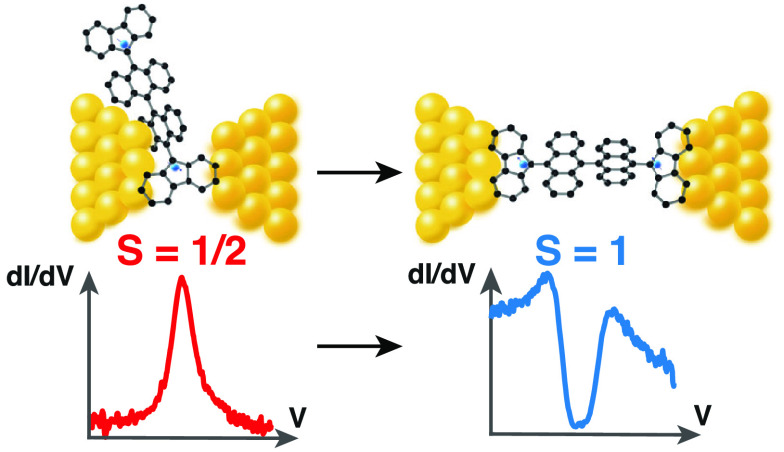

Polycyclic aromatic hydrocarbons radicals are organic molecules with a nonzero total magnetic moment. Here, we report on charge-transport experiments with bianthracene-based radicals using a mechanically controlled break junction technique at low temperatures (6 K). The conductance spectra demonstrate that the magnetism of the diradical is preserved in solid-state devices and that it manifests itself either in the form of a Kondo resonance or inelastic electron tunneling spectroscopy signature caused by spin-flip processes. The magnetic fingerprints depend on the exact configuration of the molecule in the junction; this picture is supported by reference measurements on a radical molecule with the same backbone but with one free spin, in which only Kondo anomalies are observed. The results show that the open-shell structures based on the bianthracene core are interesting systems to study spin–spin interactions in solid-state devices, and this may open the way to control them either electrically or by mechanical strain.

Over the last few years, atomically precise synthesis of radicals involving polycyclic aromatic hydrocarbons (PAHs) has gained attention.^2−4^ These open-shell graphene nanostructures exhibit π-paramagnetism which leads to more delocalized, mobile, and isotropic spin states than those generated by electrons in, e.g., d or f-orbitals. These newly available structures thus emerge as an ideal solution to combine spin-transport properties with large diffusion length and long coherence time. Taking advantage of these properties, organic radicals may play a key role for developing a new generation of low-power devices^5^ using spin polarization instead of charge as information carrier, storage, and processing.

**Figure 1:**
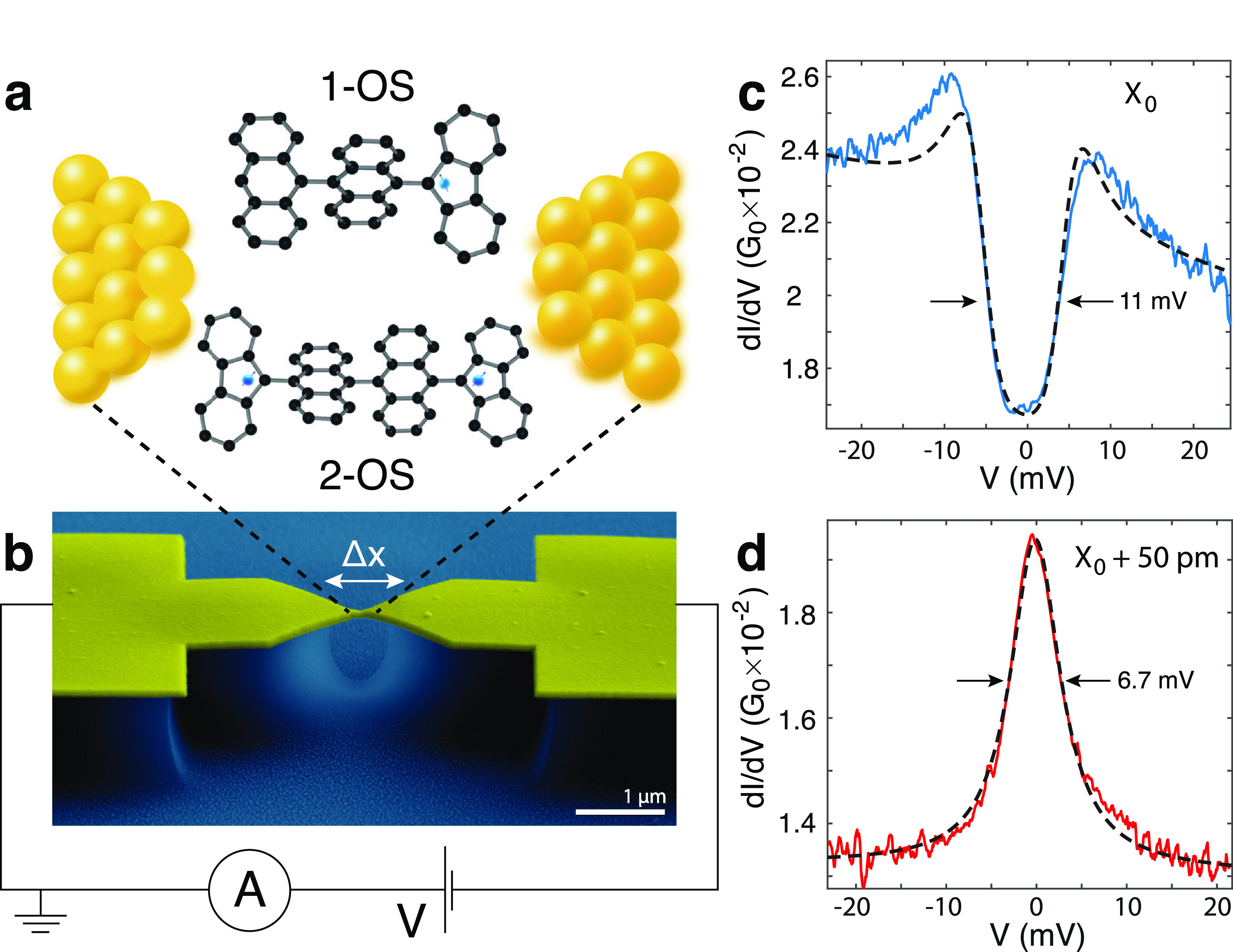
(a) Schematics of the single radical **1-OS** (upper drawing) and the diradical **2-OS** (lower drawing) in between the electrodes of a MCBJ. (b) Colored scanning electron microscopy picture of a MCBJ device. Electronic transport across the molecule is measured by sweeping the bias voltage across the electrodes while measuring the resulting current. (c,d) Typical molecular features observed in the differential conductance (d*I*/d*V*) of a **2-OS** molecule (breaking series XIV, see SI Table S1): (c) a dip around zero-bias caused by an inelastic spin-flip excitation (dotted line: fit to the data using the model of ref (1)) and (d) a peak centered around zero-bias with a full width half-maximum (fwhm) of 6.7 mV. The Kondo resonance (d) is obtained by increasing the distance between the electrodes from an initial position (*x*_0_) in (c) by 50 pm.

In single-molecule transport studies, the magnetic signatures of a radical are typically revealed by the observation of Kondo resonances.^6^ For example, previous scanning tunneling microscopy and break junction experiments focused on all-organic radicals^7^ display Kondo temperatures (*T*_K_) ranging from a few^8^ K up to tens^9−11^ of K. Studies on other charge transport phenomena such as magnetoresistance^12,13^ and magnetic-field induced variation of inelastic electron tunneling spectroscopy (IETS) have also been reported.^14,15^ Using IETS, spin–spin interactions in all organic diradicals have been studied, demonstrating interesting properties such as large exchange couplings^16,17^ or ground-state inversion by electrostatic gating.^18^ Here, we report on measurements of all-organic mono- and diradical molecules in a mechanically controlled break junction (MCBJ). For this study, we selected bianthracene-based radicals **1-OS** and **2-OS** (see Figure 2), which are considered as stable open-shell PAHs due to the steric hindrance around the radical centers.^19^ For both molecules, Kondo resonances were observed with Kondo temperatures ranging from 7 to 50 K; only for the diradical IETS signals are found that reveal spin-flip excitation with exchange energies of about 10 mV.

**Figure 2:**
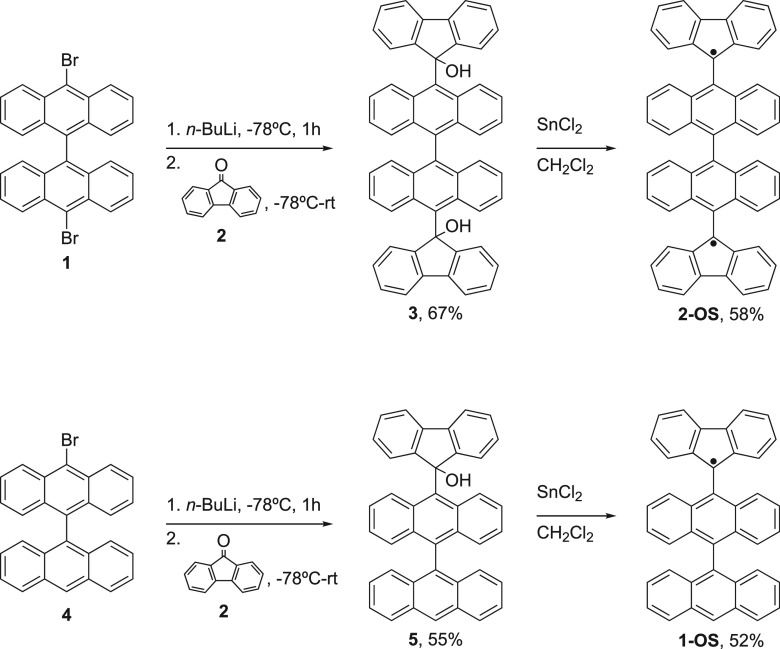
Synthesis of bianthracene-based radicals **2-OS** (top) and **1-OS** (bottom).

We synthesized the diradical **2-OS** following the procedure described by Zeng et al.^19^ (see Figure 2). In particular, 10,10′-dibromo-9,9′-bianthracene (**1**) was sequentially treated with *n*-BuLi and fluorenone (**2**) to afford diol (**3**). Then, reduction of this compound with SnCl_2_ led to the formation of diradical **2-OS** which was stable enough to be purified by column chromatography and isolated under ambient conditions. A similar synthetic procedure was used for the preparation of the monoradical **1-OS** starting from 10-bromo-9,9′-bianthracene (**4**). In this case, compound **1-OS** was isolated as a red solid in 52% yield by reduction of alcohol **5** with SnCl_2_.

The diradical **2-OS** is composed of two fluorene moieties each hosting a C atom with an unpaired electron, linked by two anthracene units. The monoradical version **1-OS** has the same structure without one of the fluorenes ends. Both molecules do not host sulfur-based linker groups to stabilize the binding with the electrodes; the electrode–molecule contacts thus rely on van der Waals interactions between the aromatic cycles and the gold atoms of the electrodes or the formation of Au–C bonds.^20^

We employed lithographically defined mechanically controlled break junctions (MCBJs) to measure the conductance of **1-OS** and **2-OS**. The substrate with the device is held in a three-point bending mechanism at the bottom of a cryogenic stick in a vacuum chamber; measurements are performed at low temperature by plunging the stick in a liquid helium bath (*T* ≈ 7 K) or in some cases in a helium dewar with a 8 T magnet. The electronic transport characteristics are measured in a two-probe scheme by applying a small voltage, *V*, and reading out the current, *I* (see Figure 1b). The differential conductance d*I*/d*V* is then computed numerically with a Savitzky–Golay filter (Figure 1c,d).

We prepared a solution at ambient conditions from a small quantity of **1-OS** or **2-OS** in powder form diluted in dichloromethane (DCM) at approximately a 0.1 mM concentration. The solution is then dropcasted on the devices, and the chamber of the dip stick containing the sample space is pumped to evaporate the solvent. Subsequently, the sample is cooled down by putting the dip stick in a vessel with liquid helium. The junction is then broken rapidly to the point where the gold atoms no longer link the two sides of the electrodes (we chose this point to be *G* < 0.3*G*_0_), where *G*_0_ = 2*e*^2^/ℏ is the conductance quantum with *e* the electron charge and ℏ Planck’s constant. Afterward, the gap spacing is increased in small steps of about 5 pm and current–voltage characteristics (*IV*’s) are acquired at each step. The conductance of the junction is measured while separating the electrodes until the current drops below the noise level of 10^–6^*G*_0_. The electrodes are then pushed back together, and the procedure is repeated to statistically assess different molecule-metal configurations. The *IV*’s acquired during one stretching event belong to the same breaking trace.

We measured 2114 different breaking traces in five different samples with **2-OS**. In total, 34805 *IV*’s were taken from which two molecule features were identified in the d*I*/d*V* spectra: A zero-bias peak (Figure 1d) and an inelastic electron tunneling spectroscopy (IETS; Figure 1c) feature with a clear suppression of the current around zero bias. Over all devices measured, 55 junctions (about 2.7% of the total) showed a zero-bias peak and 18 (about 1%) showed an IETS signal with current suppression around zero-bias with a flat region around *V* = 0. We note that most junctions do not show molecular features either in the *IV*’s or breaking traces: More than 50% of breaking traces showed an exponential decrease of the zero-bias conductance with gap spacing, consistent with direct, single barrier tunneling without a molecule.

We assign the zero-bias resonance peaks to a Kondo resonance and fit a Lorentzian function to them (Figure 1d):
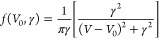
1where *V*_0_ is the resonance center taken to be zero and 2γ is the full width half-maximum (fwhm). The fwhm is used to approximate^6^ the Kondo temperature, *T*_K_:
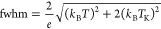
2where *k*_B_ is the Boltzmann constant, and *T* is the sample temperature. We apply this fitting to each d*I*/d*V* with a zero-bias peak, and the results for the fwhm are summarized in Figure 3a. Most fwhm’s lie between 4 and 10 meV corresponding, respectively, to a Kondo temperature of 7 and 50 K; the variation indicates that the electronic coupling between the spin and the metal electrode can differ substantially. A Kondo temperature of a few tenths of K is typical for resonances observed in molecular devices^21,22^ but is large compared to values found for spin-1 molecules.^23^

**Figure 3:**
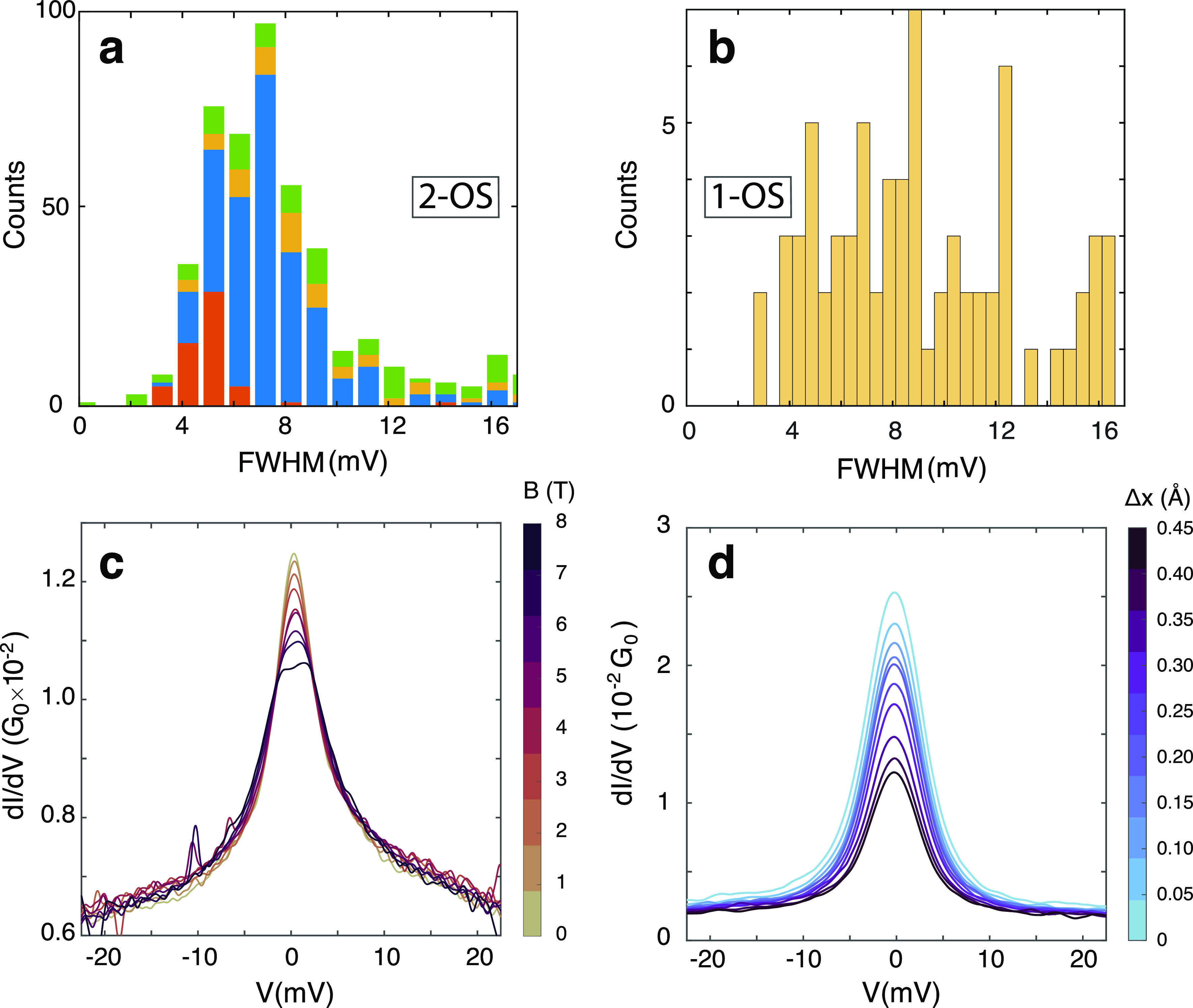
(a) Statistics on the widths of the zero-bias peaks of the **2-OS** diradical molecule as determined from a fit of a Lorentzian function to the d*I*/d*V* spectra. Each color corresponds to a different sample. The Kondo temperature is approximated from the extracted fwhm using eq 2. The average width is 7 mV corresponding to a Kondo temperature of 34 K. (b) Same statistics as in (a) for the **1-OS** monoradical reference measurements showing a smaller number of peaks with a wider distribution. (c) Magnetic field dependence of a zero-bias peak which has *T*_K_(*B* = 0) = 30 K (measurement performed on an **2-OS** molecule, offset vertically for clarity). (d) Mechanical manipulation of a Kondo resonance measured on an **2-OS** molecule with a peak initial width at *x* = *x*_0_ of 6.9 mV (*T*_K_ ≈ 33.5 K). The peak width and Kondo temperature reduce when increasing the spacing between the electrodes: Breaking the electrodes 0.5 Å further apart yields a peak width of 6.4 mV (*T*_K_ ≈ 30 K).

We also measured 4235 breaking traces in the presence of the **1-OS** single radical (see Figure 1a). We observed zero-bias peaks in 107 breaking traces (about 2.5%). A fraction of the resonances had a fwhm value similar to that of the **2-OS** molecule (see Figure 3b); some zero-bias features, however, exhibit a larger fwhm in the range of 20–30 mV. Most likely, these do not originate from Kondo physics, and we speculate that they correspond to transport involving a nearby resonance (see SI, Section II-c).

When applying a magnetic field, *B*, one expects the Zeeman energy to compete with the Kondo temperature, thereby lifting the degeneracy of the spin states involved in transport through the molecule. This competition leads to a splitting of the zero-bias resonance by approximately 2*g*μ_B_*B* where *g* = 2 is the *g*-factor of the electron spin and μ_B_ is the Bohr magneton. In the strong coupling limit (*T* ≪ *T*_K_), this splitting is expected to take place beyond the critical field, *B*_c_ given by^6,24,25^
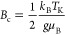
3

Applying this formula to the Kondo resonance displayed in Figure 3c, *B*_c_ would be 11 T. In our setup, the maximum magnetic field available is *B* = 8 T, and with this field, we could not split the Kondo resonance, consistent with the single spin-(1/2) Kondo physics discussed (eq 2).

Typically, a single breaking trace shows a few *IV*’s with zero-bias anomalies; in some cases, the junction is more stable during stretching of the device allowing the zero-bias anomaly to be measured over distances up to 0.5 nm. An example is shown in Figure 3d where the width and height of the peak decrease when the separation increases between the electrodes. At the same time, the average conductance background of the spectra also decreases because direct tunneling between the electrodes is reduced. In Figure 3d, the fwhm decreases by about 10%, whereas the height decreases by about 50%. This behavior is consistent with a lowering of one or both electronic molecule–electrode couplings when moving the electrodes apart.

We now turn the analysis to the step-like increases of the conductance in the d*I*/d*V* spectra (Figure 1c). These signals indicate the opening of an inelastic spin-flip tunneling channel via an excited state of the molecule. For the **2-OS** system, this is the singlet–triplet excitation with the corresponding energy scale, *J*_ex_, of the two-spin system formed by the unpaired electrons of the diradical molecule. We fit the Ternes model,^1^ a model commonly used in the scanning tunneling microscopy community, to the data as shown in Figure 1c (fits are the dotted lines). We note that these IETS spectra were not observed for the **1-OS** molecule, pointing to the importance of the presence of two spins for its observation.

We have extracted *J*_ex_ from the fits of the IETS signals and plot their absolute values in Figure 4a; see SI for additional spectra and the fit parameters (Table S1). Most counts are around an energy of 11 meV, close to the value found in SQUID magnetometry.^19^ The IETS signal from Figure 4b was stable enough for magnetic field measurements. The magnetic field lifts the degeneracy of the triplet state and changes the excitation energy depicted in the inset of Figure 4c. The lifting of degeneracy initially suppresses the small zero-bias contribution, and a step appears around zero-bias above 3 T. The same data are shown in the colormap of Figure 4c, where the corresponding transitions are depicted by white arrows. The transitions correspond to an open-shell triplet (*S* = 1) ground state with a singlet (*S* = 0) excited state (ferromagnetic coupling). This behavior is well captured by the Ternes model (dotted lines of Figure 4b), but in the data, a larger suppression of the current around zero-bias is present compared to numerical simulations (more details can be found in SI section II-b).

**Figure 4:**
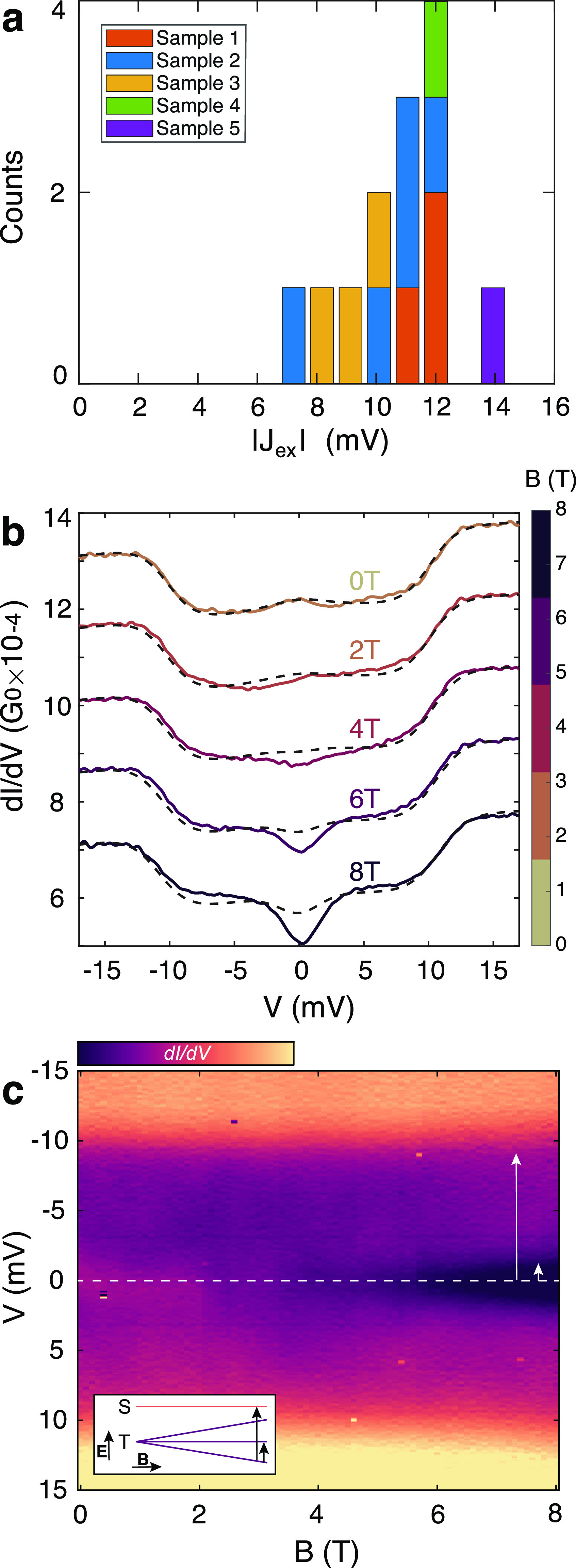
(a) Histogram of the exchange coupling values determined from *IV*’s displaying an IETS signal. Most of the observed transitions are around 10 mV, close to the value measured in the bulk using SQUID magnetometry.^19^ (b) Measured inelastic tunneling spectra (solid colored lines) are fitted to the Ternes model^1^ (black dashed lines) at different values of the applied magnetic field (**2-OS** molecular breaking series I, see Table S1 in SI for the fit parameters). The step opening at zero bias corresponds to a new transition allowed by lifting the degeneracy of the triplet ground state. (c) Colormap of the magnetic field sweep corresponding to (b). Inset shows the evolution in a magnetic field of the energy of the triplet (T) and singlet (S) states; black arrows correspond to the transitions indicated by white arrows in the main panel.

We observe a range of singlet–triplet energy gaps ranging from 7 to 14 meV. The magnetic field measurement from Figure 4b,c shows that the exchange coupling of the spins in this device is ferromagnetic. Within the Ternes model, the small zero-bias enhanced conductance is also explained by having a triplet ground state due to higher-order Kondo contributions. On the other hand, two IETS spectra show a conductance overshoot at the step-edges in the absence of zero-bias increases (as in Figure 1c). These overshoots may be an indication of an antiferromagnetic ground state (no magnetic field measurements were performed on these samples) and are further discussed in Supporting Information section II-b.

In the **2-OS** molecule, the two anthracene units linking each moieties hosting an unpaired electron have a rotational degree of freedom that impacts the overlap of the two unpaired electron wave functions.^26^ This effect can result in different values of the exchange coupling between them. The hybridization with the electrodes producing the Kondo resonances can also contribute to the delocalization of the unpaired electrons and thus modify this overlap. A variation of the exchange coupling and change of sign have been observed in other organic radicals^18^ where both ferromagnetic and antiferromagnetic ground states were found, indicating that the conformation of the molecule is important in determining the spin–spin interactions on it. The large magnitude of the exchange coupling in the **2-OS** molecule is in contrast with previous measurements where typical values are 1 order of magnitude lower.^27,28^ Similar values of the exchange coupling in an *S* = 1 diradical system have been reported for a triangulene dimer,^16^ highlighting the magnetic properties of free electrons in carbon lattices.

The two magnetic fingerprints discussed depend on the molecule-electrode geometry. The geometry defines the charge injection points into the molecule (see Figure 5). The absence of specific anchoring groups may allow for a larger range of electronic coupling in the junction compared to sulfur-anchoring group containing molecules. Configurations with strong molecule–electrode coupling involving transport across one of the unpaired spins induce *S* = 1/2 Kondo resonances in the d*I*/d*V* spectra. With a small gap between the electrodes, several configurations may involve electron pathways interacting mainly with one of the radical centers. Fewer configurations of the molecule bridging the electrodes are possible at larger distances favoring an extended molecule configuration; electrons flowing through the molecule in this configuration interact with both radical centers, leading to spin-flip *S* = 1 features in the spectra.

**Figure 5:**
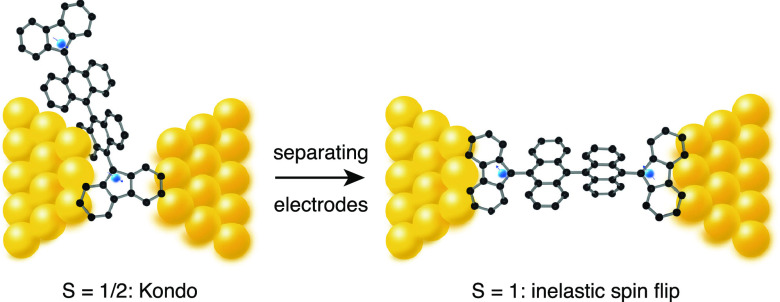
At small electrode distances, the geometry of the molecule in the junction allows injection at several points into the molecule; some configurations may only involve transport across one radical center giving rise to spin-(1/2) effects in the d*I*/d*V* spectrum. At larger distances between the electrodes, fewer configurations of the molecule bridging the two electrodes are possible. This favors an extended molecule configuration in the gap as shown on the right. The electrons flowing across the junction interact with both radical centers, leading to spin-flip features in the differential conductance spectrum.

In summary, we contacted PAH radical molecules in solid-state devices. We show that the free electrons of these radicals are preserved after integration in mechanically controlled break junctions and the open-shell character persists against mechanical manipulation. We observed spin-1/2 Kondo resonances in the electronic transport spectra of both the mono- and diradicals and spin-1 inelastic spin-flip effects for the **2-OS** devices only. The exchange coupling of the diradical was found to be in the range of 10 meV with predominantly a triplet ground-state. The MCBJ technique allows to probe different molecule–metal configurations so that an extended picture of the behavior of the radical molecules in a solid-state device is obtained. Understanding the associated variations in parameters is important for future use in these devices. From a fundamental point of view, the robustness of the radicals and the different physical effects highlight these systems as excellent models for the investigation of magnetic effects on electronic transport at the single-molecule scale.

## Notes

The complete data set of the MCBJ experiments with description and code are available free of charge.

## Supporting Information Available

Synthetic details: General methods for the synthesis of diradical **2-OS** and monoradical **1-OS**, synthesis of alcohol, syntheis of the monoradical **1-OS**, and spectroscopic data. Additional data: Reference measurement, fit parameters of IETS spectra for **2-OS**, and **1-OS** monoradical (PDF)

## References

[ref1] Ternes M. Spin excitations and correlations in scanning tunneling spectroscopy. New J. Phys. 2015, 17, 063016 10.1088/1367-2630/17/6/063016.

[ref2] Pavliček N.; Mistry A.; Majzik Z.; Moll N.; Meyer G.; Fox D. J.; Gross L. Synthesis and characterization of triangulene. Nat. Nanotechnol. 2017, 12, 308–311. 10.1038/nnano.2016.305. 28192389

[ref3] Chen Z.; Narita A.; Müllen K. Graphene Nanoribbons: On-Surface Synthesis and Integration into Electronic Devices. Adv. Mater. 2020, 32, 2001893 10.1002/adma.202001893. 32945038

[ref4] Zhang X.; Xu Z.; Si W.; Oniwa K.; Bao M.; Yamamoto Y.; Jin T. Synthesis of extended polycyclic aromatic hydrocarbons by oxidative tandem spirocyclization and 1,2-aryl migration. Nat. Commun. 2017, 8, 15073 10.1038/ncomms15073. 28440319 PMC5414065

[ref5] Hirohata A.; Yamada K.; Nakatani Y.; Prejbeanu I.-L.; Diény B.; Pirro P.; Hillebrands B. Review on spintronics: Principles and device applications. J. Magn. Magn. Mater. 2020, 509, 166711 10.1016/j.jmmm.2020.166711.

[ref6] Scott G. D.; Natelson D. Kondo Resonances in Molecular Devices. ACS Nano 2010, 4, 3560–3579. 10.1021/nn100793s. 20568709

[ref7] Zhang Y.-h.; Kahle S.; Herden T.; Stroh C.; Mayor M.; Schlickum U.; Ternes M.; Wahl P.; Kern K. Temperature and magnetic field dependence of a Kondo system in the weak coupling regime. Nat. Commun. 2013, 4, 2110 10.1038/ncomms3110. 23817525 PMC3730050

[ref8] Frisenda R.; Gaudenzi R.; Franco C.; Mas-Torrent M.; Rovira C.; Veciana J.; Alcon I.; Bromley S. T.; Burzurí E.; van der Zant H. S. J. Kondo Effect in a Neutral and Stable All Organic Radical Single Molecule Break Junction. Nano Lett. 2015, 15, 3109–3114. 10.1021/acs.nanolett.5b00155. 25897770

[ref9] Yu L. H.; Natelson D. The Kondo effect in C60 single-molecule transistors. Nano Lett. 2004, 4, 79–83. 10.1021/nl034893f.

[ref10] Liu J.; Isshiki H.; Katoh K.; Morita T.; Breedlove B. K.; Yamashita M.; Komeda T. First observation of a Kondo resonance for a stable neutral pure organic radical, 1, 3, 5-triphenyl-6-oxoverdazyl, adsorbed on the Au (111) surface. J. Am. Chem. Soc. 2013, 135, 651–658. 10.1021/ja303510g. 23240646

[ref11] Mullegger S.; Rashidi M.; Fattinger M.; Koch R. Surface-supported hydrocarbon π radicals show Kondo behavior. J. Phys. Chem. C 2013, 117, 5718–5721. 10.1021/jp310316b. PMC3607337 23539333

[ref12] Hayakawa R.; Karimi M. A.; Wolf J.; Huhn T.; Zollner M. S.; Herrmann C.; Scheer E. Large magnetoresistance in single-radical molecular junctions. Nano Lett. 2016, 16, 4960–4967. 10.1021/acs.nanolett.6b01595. 27458666

[ref13] Warner B.; El Hallak F.; Prüser H.; Sharp J.; Persson M.; Fisher A. J.; Hirjibehedin C. F. Tunable magnetoresistance in an asymmetrically coupled single-molecule junction. Nature Nanotechnol. 2015, 10, 259–263. 10.1038/nnano.2014.326. 25622229

[ref14] Gaudenzi R.; De Bruijckere J.; Reta D.; Moreira I. D. P.; Rovira C.; Veciana J.; Van Der Zant H. S.; Burzurí E. Redox-induced gating of the exchange interactions in a single organic diradical. ACS Nano 2017, 11, 5879–5883. 10.1021/acsnano.7b01578. 28494146 PMC5492214

[ref15] Chen X.; Fu Y.-S.; Ji S.-H.; Zhang T.; Cheng P.; Ma X.-C.; Zou X.-L.; Duan W.-H.; Jia J.-F.; Xue Q.-K. Probing Superexchange Interaction in Molecular Magnets by Spin-Flip Spectroscopy and Microscopy. Phys. Rev. Lett. 2008, 101, 197208 10.1103/PhysRevLett.101.197208. 19113306

[ref16] Mishra S.; Beyer D.; Eimre K.; Ortiz R.; Fernández-Rossier J.; Berger R.; Gröning O.; Pignedoli C. A.; Fasel R.; Feng X.; Ruffieux P. Collective All-Carbon Magnetism in Triangulene Dimers**. Angew. Chem., Int. Ed. 2020, 59, 12041–12047. 10.1002/anie.202002687. PMC7383983 32301570

[ref17] Mishra S.; Yao X.; Chen Q.; Eimre K.; Gröning O.; Ortiz R.; Di Giovannanto-nio M.; Sancho-García J. C.; Fernández-Rossier J.; Pignedoli C. A.; et al. Large magnetic exchange coupling in rhombus-shaped nanographenes with zigzag periphery. Nat. Chem. 2021, 13, 581–586. 10.1038/s41557-021-00678-2. 33972756

[ref18] Gaudenzi R.; Burzurí E.; Reta D.; Moreira I. d. P. R.; Bromley S. T.; Rovira C.; Veciana J.; van der Zant H. S. J. Exchange Coupling Inversion in a High-Spin Organic Triradical Molecule. Nano Lett. 2016, 16, 2066–2071. 10.1021/acs.nanolett.6b00102. 26862681

[ref19] Zeng Z.; et al. Stable Tetrabenzo-Chichibabin’s Hydrocarbons: Tunable Ground State and Unusual Transition between Their Closed-Shell and Open-Shell Resonance Forms. J. Am. Chem. Soc. 2012, 134, 14513–14525. 10.1021/ja3050579. 22889277

[ref20] Poli R. Radical Coordination Chemistry and Its Relevance to MetalMediated Radical Polymerization. Eur. J. Inorg. Chem. 2011, 2011, 1513 10.1002/ejic.201001364.

[ref21] Park J.; Pasupathy A. N.; Goldsmith J. I.; Chang C.; Yaish Y.; Petta J. R.; Rinkoski M.; Sethna J. P.; Abruña H. D.; McEuen P. L.; Ralph D. C. Coulomb blockade and the Kondo effect in single-atom transistors. Nature 2002, 417, 722–725. 10.1038/nature00791. 12066179

[ref22] Scott G. D.; Keane Z. K.; Ciszek J. W.; Tour J. M.; Natelson D. Universal scaling of nonequilibrium transport in the Kondo regime of single molecule devices. Phys. Rev. B 2009, 79, 165413 10.1103/PhysRevB.79.165413.

[ref23] Parks J. J.; Champagne A. R.; Costi T. A.; Shum W. W.; Pasupathy A. N.; Neuscamman E.; Flores-Torres S.; Cornaglia P. S.; Aligia A. A.; Balseiro C. A.; Chan G. K.-L.; Abruña H. D.; Ralph D. C. Mechanical Control of Spin States in Spin-1 Molecules and the Underscreened Kondo Effect. Science 2010, 328, 1370–1373. 10.1126/science.1186874. 20538943

[ref24] Costi T. A. Kondo Effect in a Magnetic Field and the Magnetoresistivity of Kondo Alloys. Phys. Rev. Lett. 2000, 85, 1504–1507. 10.1103/PhysRevLett.85.1504. 10970540

[ref25] Meir Y.; Wingreen N. S.; Lee P. A. Low-temperature transport through a quantum dot: The Anderson model out of equilibrium. Phys. Rev. Lett. 1993, 70, 2601–2604. 10.1103/PhysRevLett.70.2601. 10053604

[ref26] Venkataraman L.; Klare J. E.; Nuckolls C.; Hybertsen M. S.; Steigerwald M. L. Dependence of single-molecule junction conductance on molecular conformation. Nature 2006, 442, 904–907. 10.1038/nature05037. 16929295

[ref27] Ormaza M.; Bachellier N.; Faraggi M. N.; Verlhac B.; Abufager P.; Ohresser P.; Joly L.; Romeo M.; Scheurer F.; Bocquet M.-L.; Lorente N.; Limot L. Efficient Spin-Flip Excitation of a Nickelocene Molecule. Nano Lett. 2017, 17, 1877–1882. 10.1021/acs.nanolett.6b05204. 28199115

[ref28] Jo M.-H.; Grose J. E.; Baheti K.; Deshmukh M. M.; Sokol J. J.; Rumberger E. M.; Hendrickson D. N.; Long J. R.; Park H.; Ralph D. C. Signatures of Molecular Magnetism in Single-Molecule Transport Spectroscopy. Nano Lett. 2006, 6, 2014–2020. 10.1021/nl061212i. 16968018

