# Supplementary material for: Magnetic Fingerprints in an All-Organic Radical Molecular Break Junction
Source: Nano Lett. 2022 Oct 7;22(20):8086–92. doi: 10.1021/acs.nanolett.2c02326 (PMC9614975; doi:10.1021/acs.nanolett.2c02326)
Supplement: Supplementary file 1 — nl2c02326_si_001.pdf [file nl2c02326_si_001.pdf]

Supporting information: Magnetic fingerprints in an all-organic radical molecular break junction

Thomas Y. Baum,*,† Saleta Fernández,‡ Diego Peña,*,‡ and Herre S. J. van der Zant*,†

†*Kavli Institute of Nanoscience, Delft University of Technology, Lorentzweg 1, 2628 CJ Delft, The Netherlands*

‡*Centro Singular de Investigación en Química Biolóxica e Materiais Moleculares (CiQUS) and Departamento de Química Orgánica, Universidade de Santiago de Compostela*

E-mail: t.y.baum@tudelft.nl; diego.pena@usc.es; h.s.j.vanderZant@tudelft.nl

Contents

I- Synthetic details **2**

a. General methods for the synthesis of diradical **2-OS** and monoradical **1-OS** 2

b. Synthesis of alcohol **5** 4

c. Synthesis of the monoradical **1-OS** 5

d. Spectroscopic data 6

II- Additional data **11**

a. Reference measurement 11

b. Fits of IETS spectra 2-OS 11

c. 1-OS monoradical 16

I- Synthetic details

a. General methods for the synthesis of diradical 2-OS and mono-radical 1-OS

All the reactions were carried out under argon using oven-dried glassware. CH_2Cl_2 and Tetrahydrofuran (THF) were dried using a MBraun SPS-800 Solvent Purification System. Finely powdered $SnCl_2$ was purchased from Sigma-Aldrich, opened and stored in a glove-box. Other commercial reagents were purchased from ABCR GmbH, Sigma-Aldrich or Acros Organics, and were used without further purification. Deuterated solvents were purchased from Acros Organics. TLC was performed on Merck silica gel 60 F254 and chromatograms were visualized with UV light (254 and 365 nm) and/or stained with Hanessian's stain. Column chromatography was performed on Merck silica gel 60 (ASTM 230-400 mesh). ^{1}H and ^{13}C NMR spectra were recorded at 300 and 75 MHz (Varian Mercury-300 instrument) or 500 and 125 MHz (Varian Inova 500 or Bruker 500) respectively. APCI high resolution mass spectra were obtained on a Bruker Microtof. The synthesis of diradical **2-OS** was performed following a known procedure, which is shown in Scheme 1.[1]

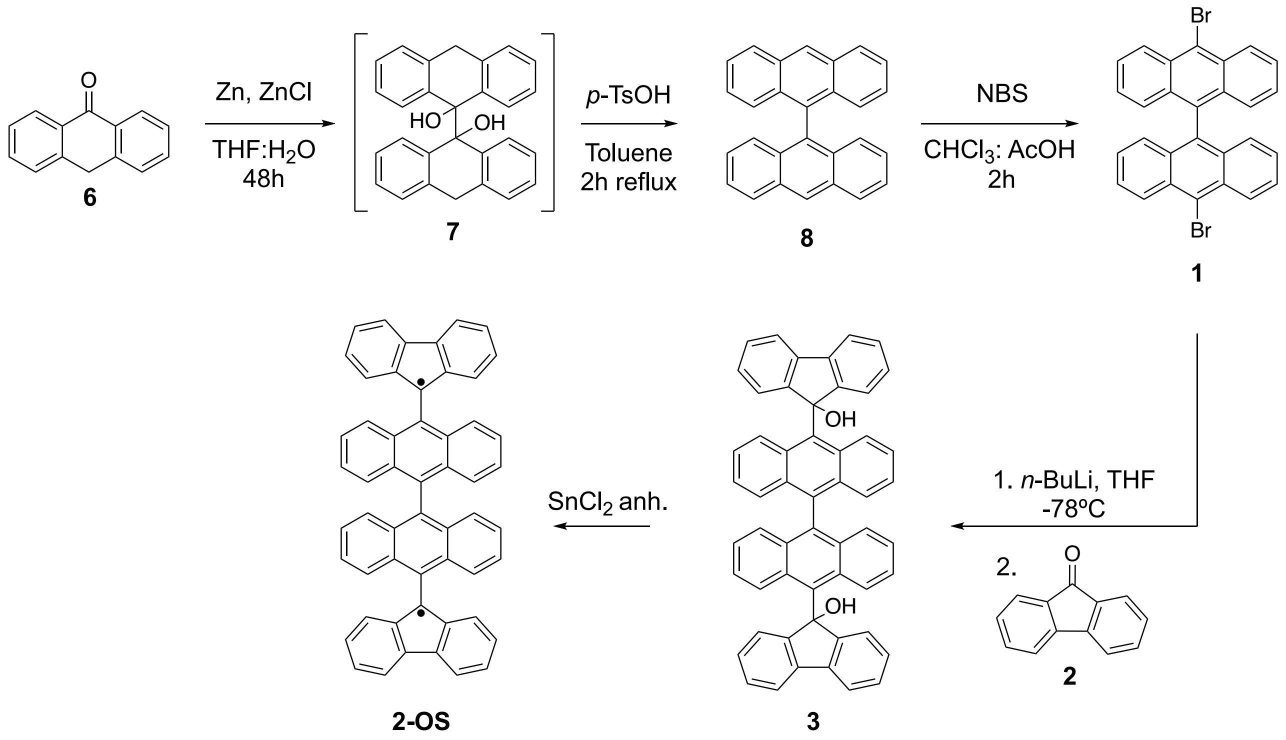

Scheme 1: Synthesis of diradical **2-OS**.

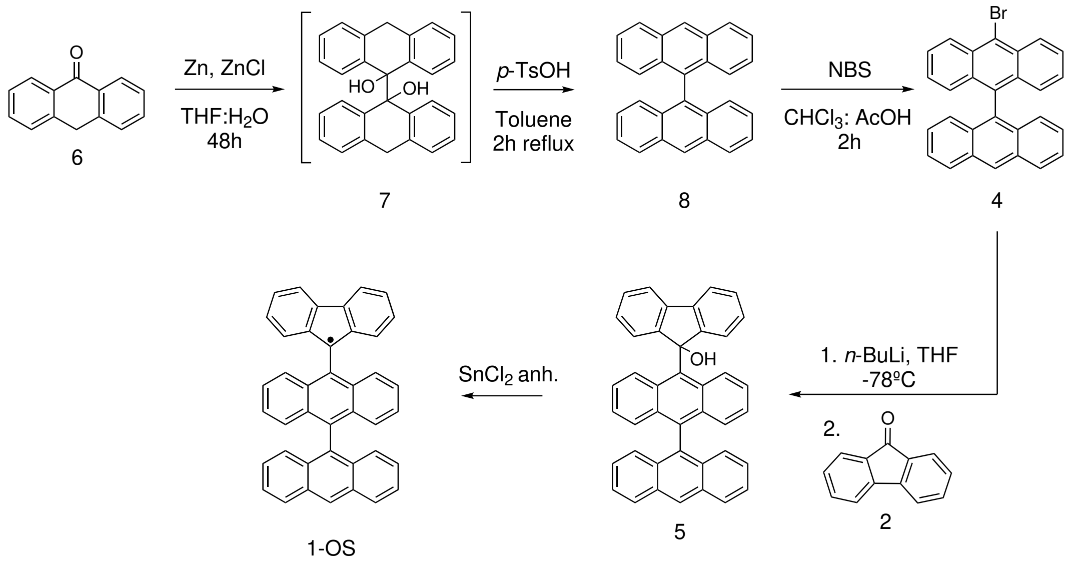

Scheme 2: Synthesis of radical **1-OS**.

The synthesis of monoradical **1-OS** was made by the route shown in Scheme 2. Compounds **1**,[2] **3**,[1] **4**[2] and **8**[3] were prepared following reported procedures.

b. Synthesis of alcohol 5

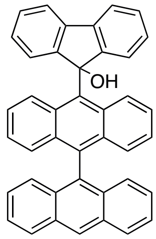

5

Scheme 3: Structure of Alcohol **5**.

In a round bottom flask compound **4** (500 mg, 0.98 mmol) was dissolved in dry THF (5 mL) under argon atmosphere. The solution was cooled to -78 °C and n-BuLi solution (2.0 M in hexane, 1.1 mL, 2.15 mmol) was slowly added. The mixture was stirred for 1h at -78 °C and then 9H-fluoren-9-one (**2**, 350 mg, 1.90 mmol) in anhydrous THF solution (5 mL) was added. The solution was slowly warmed to room temperature and stirred for 16h. Then, water (20 mL) was added and extracted with $CHCl_3$ (3x50 mL). The combined organic phases were dried over anhydrous $MgSO_4$. The solvent was removed under vacuum and the residue was purified by column chromatography (SiO_2; hexane: CH_2Cl_2 2: 1) yielding alcohol **5** (343 mg, 55%) as a yellow solid. ^{1}H NMR (300 MHz, $CDCl_3$) δ = 9.90 (d, J = 9.3 Hz, 1H), 8.68 (s, 1H), 8.15 (d, J = 8.5 Hz, 2H), 7.91 (d, J = 7.5 Hz, 2H), 7.55 – 7.40 (m, 8H), 7.32 – 7.26 (m, 1H), 7.23 – 7.07 (m, 7H), 6.98 (d, J = 8.8 Hz, 1H), 6.84-6.79 (m, 1H), 6.75-6.70 (m, 1H), 2.79 (s, 1H) ppm. ^{13}C NMR (126 MHz, $CDCl_3$) δ = 151.49 (C), 137.84 (C), 134.01 (C), 132.87 (C), 132.63 (C), 131.54(C), 130.78 (C), 130.64 (C), 130.52 (C), 130.32 (C), 128.26 (CH) 127.90 (CH), 127.51 (C), 127.34 (CH), 126.58 (CH), 126.18 (CH), 126.07 (CH), 125.94 (CH), 125.14 (CH), 124.84 (CH), 124.32 (CH), 123.98(CH), 123.58 (CH), 123.37(CH), 122.81 (CH), 120.05 (CH), 86.24 (C-OH) ppm. HRMS (APCI) calculated for $C_{41}H_{26}O$ 534.1978, found 534.1975.

c. Synthesis of the monoradical 1-OS

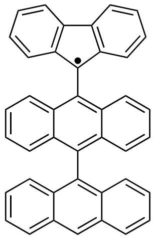

1-OS

Scheme 4: Structure of **1-OS**.

Under an argon atmosphere, a solution of **5** (150 mg, 0.28 mmol) in dry CH_2Cl_2 (6 mL) was added over $SnCl_2$ (531 mg, 2.8 mmol). The mixture was stirred 16h at room temperature. Then, the solvent was removed under vacuum and the residue was purified by flash chromatography at 0°C (SiO_2; hexane: CH_2Cl_2 3: 1) yielding **1-OS** (75 mg, 52%) as a red solid. HRMS (APCI) calculated for $C_{54}H_{32}$ $(M+H)^+$518.2029, found 518.2046.

d. Spectroscopic data

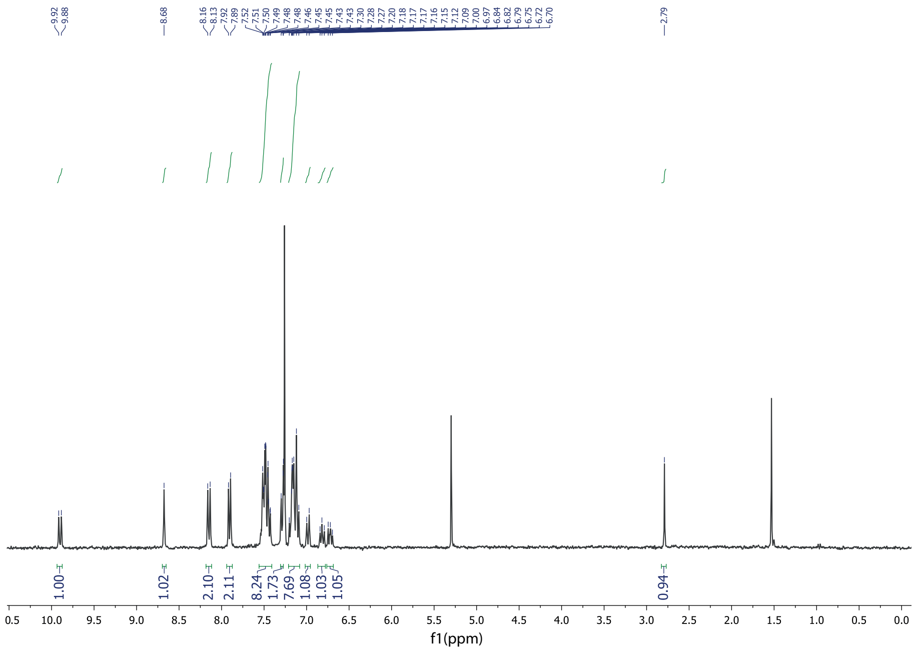

Figure S1: ^{1}H NMR (300 MHz, $CDCl_3$) of alcohol **5**.

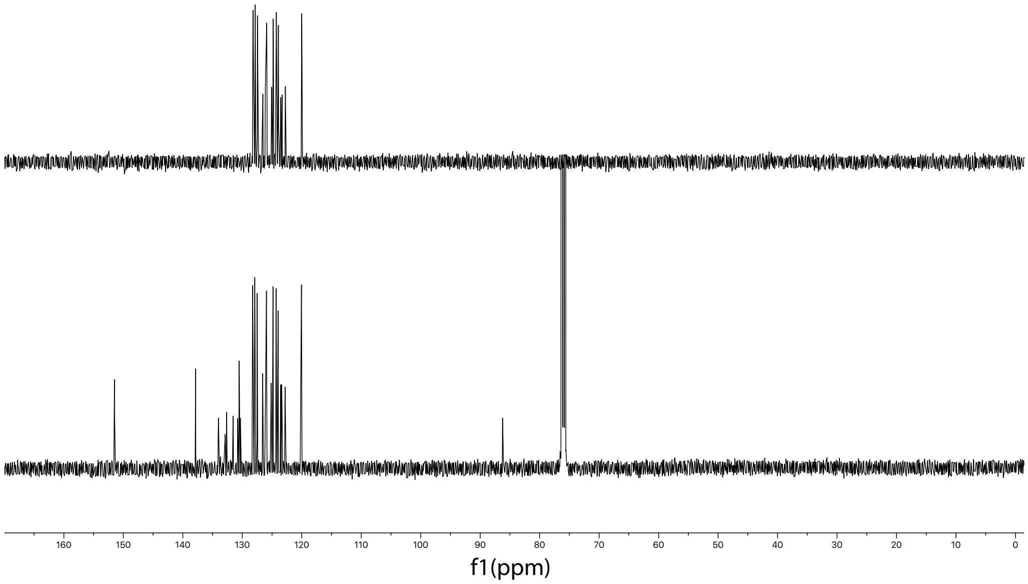

Figure S2: ^{13}C (bottom) NMR (126 MHz, $CDCl_3$) and DEPT 135 (top) of alcohol **5**.

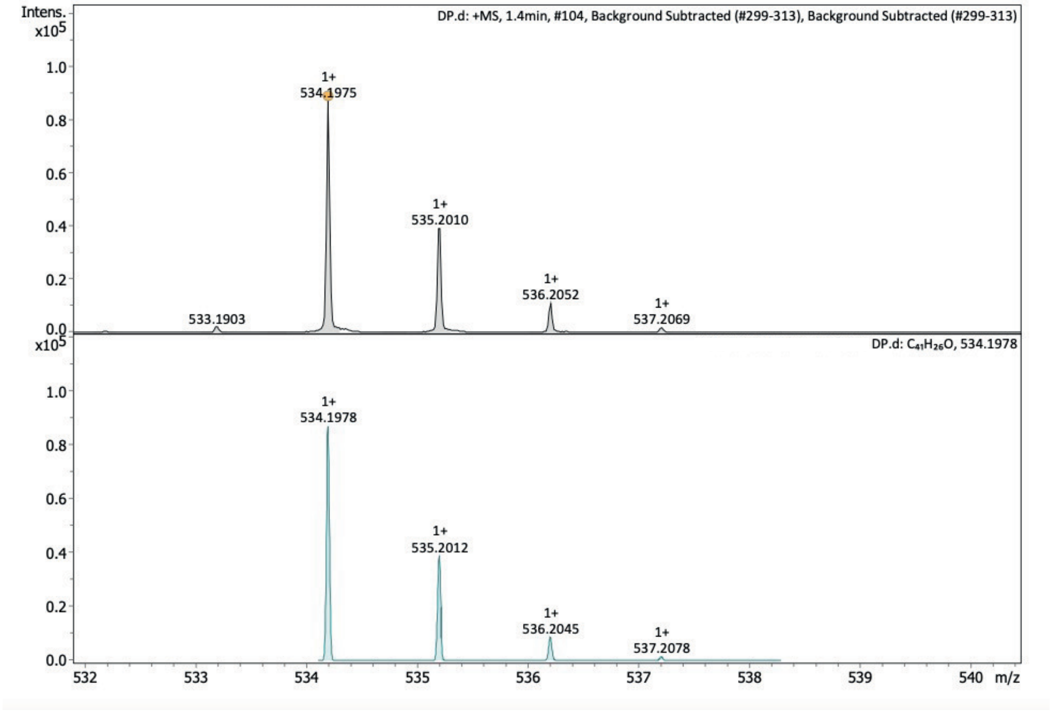

Figure S3: MS-APCI spectrum of **5** (top) and calculated isotopic distribution (bottom).

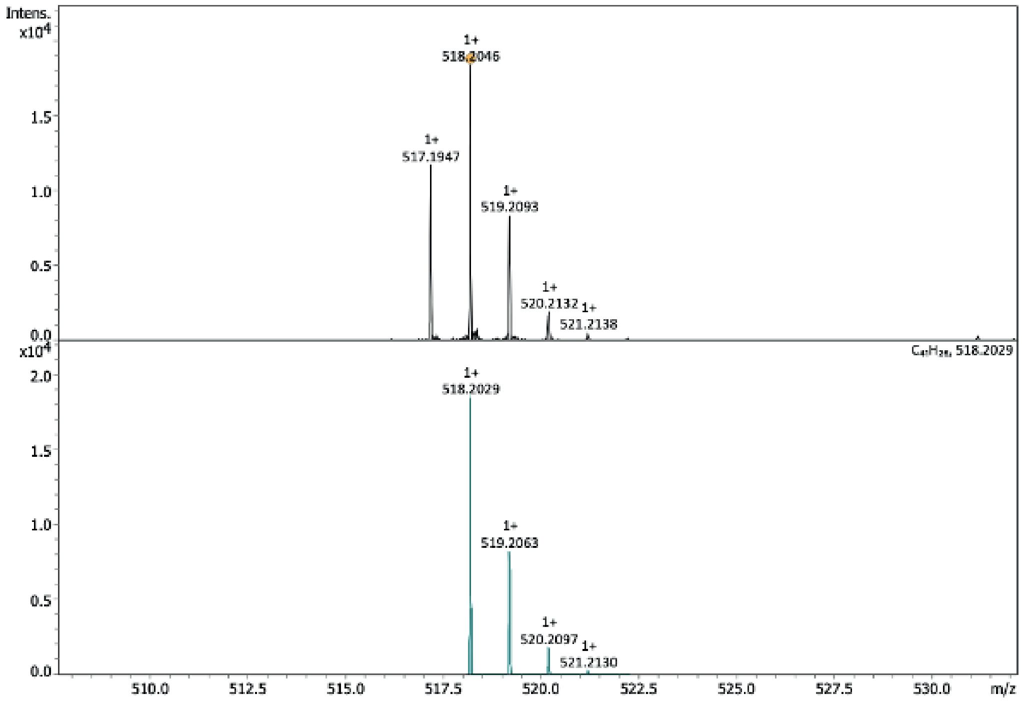

Figure S4: MS-APCI spectrum of **1-OS** (top) and calculated isotopic distribution for the [M+1] peak (bottom).

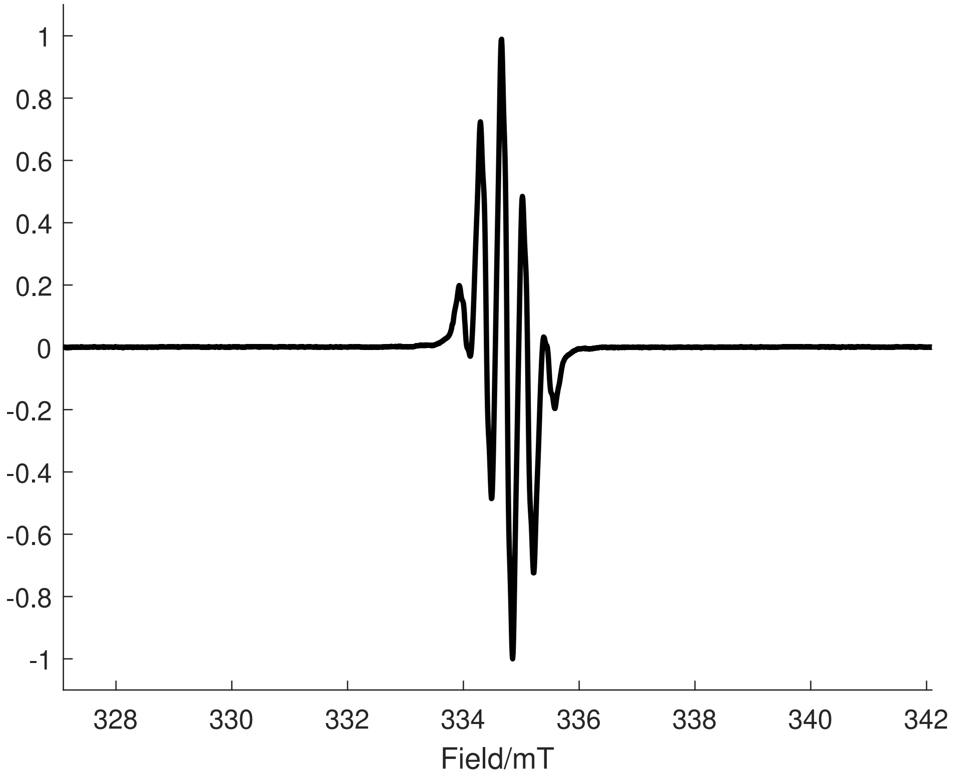

Figure S5: EPR spectrum of **1-OS** in CH_2Cl_2 solution (5x10^{-5}M) at room temperature.

In order to obtain the experimental g factor, we used the following equation:

$$g = \frac{h\nu}{\mu_{\mathrm{B}} B} \tag{1}$$

To get the correct experimental magnetic field we look at the center of the EPR spectrum and take the B in which the intensity is zero. Taking this into account and plotting the experimental values in the equation, we conclude that the experimental g factor for 1-OS is 2.0113.

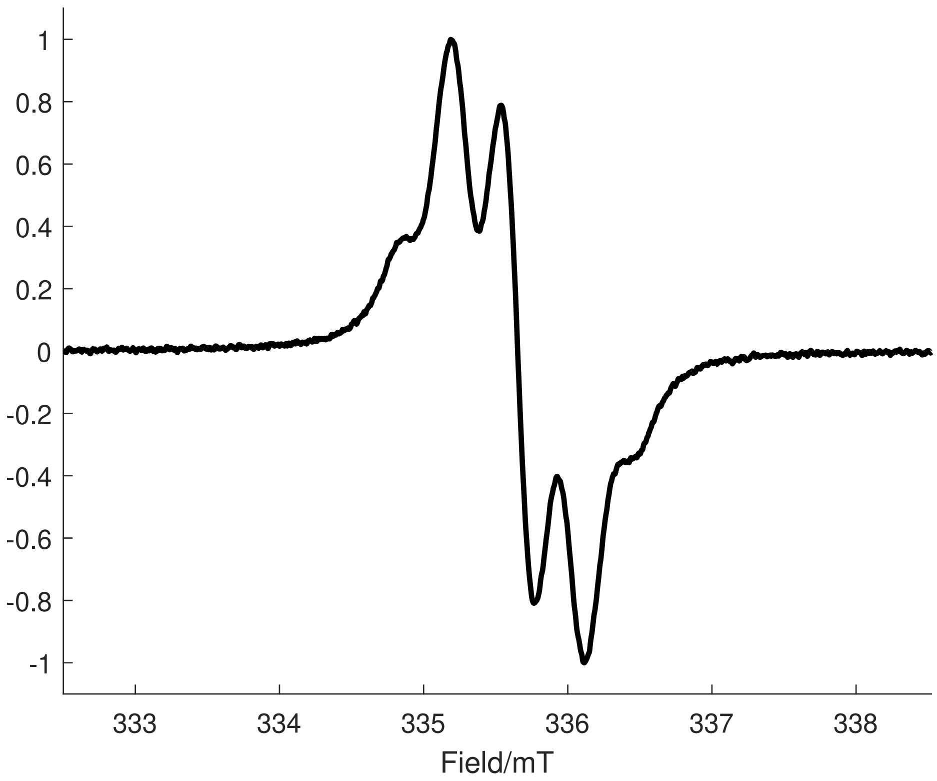

Figure S6: EPR spectrum of **2-OS** in CH_2Cl_2 solution ($5x10^{-5}$M) at room temperature.

Using equation 1, we calculate the experimental g factor for **2-OS**, obtaining a value of 2.0008.

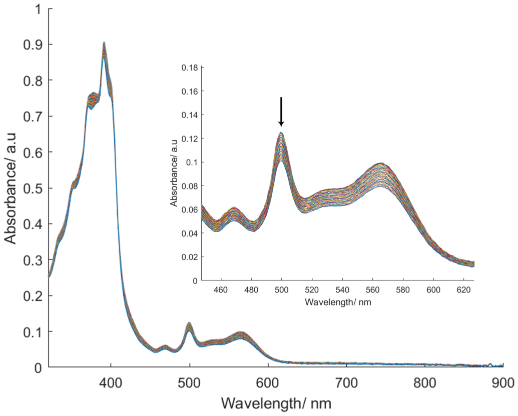

(a)

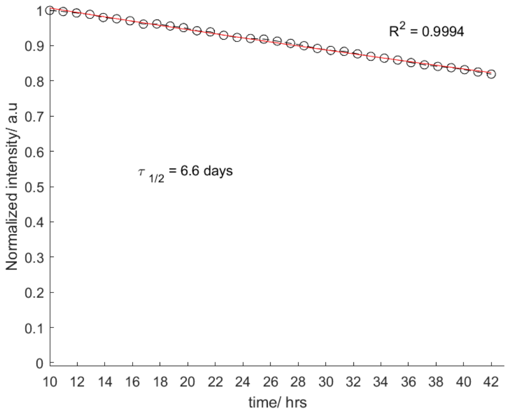

(b)

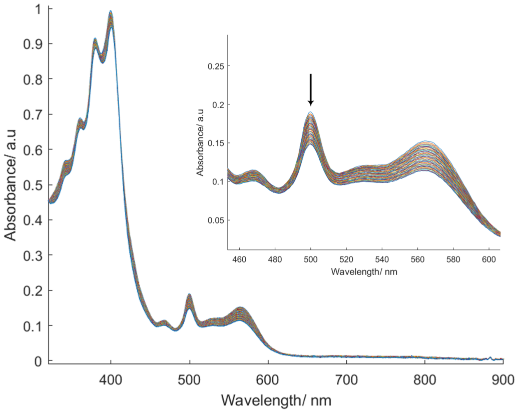

(c)

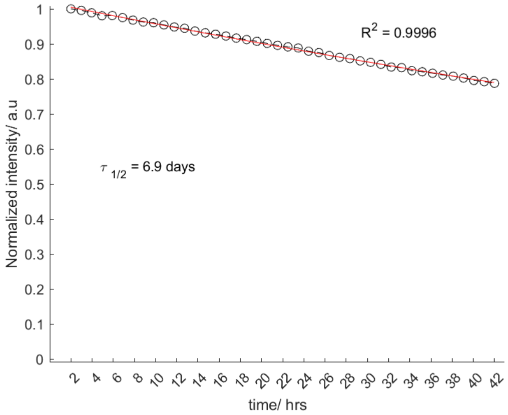

(d)

Figure S7: The UV-vis absorption spectral changes and fitting with first order kinetics at 500nm for **1-OS** (a),(b) and for **2-OS** (c),(d) in CH_2Cl_2 solution (6.4x10^{-5}M for **1-OS** and 4.9x10^{-5}M for **2-OS**) at room temperature and ambient conditions.

The half-life time of the radicals were obtained using the EasySpin package in MATLAB for first order kinectics fitting in order to solve the following equation.

$$y = A \times e^{-\mathrm{k} \times \mathrm{t}} \tag{2}$$

II- Additional data

a. Reference measurement

In the same conditions, we made reference measurements on clean mechanically controlled break junctions (at liquid helium temperature). Overall, on 600 breaking traces and 7185 IVs, none show a molecular feature in the spectra such as a zero-bias peak or an IETS-like signal.

b. Fits of IETS spectra 2-OS

We use the scilab code provided in ref.[4] to fit the IETS spectra using the following parameters in the model:

- Physical parameters
  - J_{ex}: exchange coupling (in mV)
  - T_{eff}: effective temperature (in K)
  - $J_{\rho_{\mathrm{s}}}$: Kondo scattering term
  - T_0^2: the tip-sample interaction strength (linked to the electronic coupling, Γ, in our case)
  - g: g-factor of the spin, which has been taken to be 2 for all fits
  - $B_{\mathrm{X,Y,Z}}$: magnetic field along the corresponding X, Y, Z axis (in T)
- Data offset parameters
  - b: additional background slope
  - V_{off}: additional voltage offset
  - σ_0: additional background conductance

The values of the parameters used to fit the IVs displaying an IETS signals are collected in Table **S1**. In total 14 breaking traces have been considered. For breaking series I the parameters at different magnetic fields are given. The corresponding fits are the ones shown in Fig. 4b of the main text.

Table S1: Fit parameters of the Ternes model for 14 breaking traces. Each IV fitted is labelled with its breaking series number (Roman numbers) and an integer number in case more IV are measured for the same breaking series. Δx corresponds to the electrode displacement in Ångstrom with respect to the first IV of the series (with value 0). The other fit parameters are defined in the text, see previous page.

**Breaking series**	**Fit parameters**								
I	J_{ex} (mV)	T_{eff} (K)	J_{ρ_s}	T_0^2	b	V_{off} (mV)	σ_0	Δx (Å)	inc. rates
B = 0 T	-10.14	6.8	-0.02	0.0012	0.0017	0	0.435		no
B = 1 T	-10.14	6.8	-0.02	0.0012	0.0017	0	0.435		no
B = 2 T	-10.14	6.8	-0.02	0.0012	0.0017	0	0.435		no
B = 3 T	-10.14	6.8	-0.02	0.0012	0.0017	0	0.435		no
B = 4 T	-10.14	6.8	-0.02	0.0012	0.0017	0	0.435		no
B = 5 T	-10.14	6.8	-0.02	0.0012	0.0017	0	0.435		no
B = 6 T	-10.14	6.8	-0.02	0.0012	0.0017	0	0.435		no
B = 7 T	-10.14	6.8	-0.02	0.0012	0.0017	0	0.435		no
B = 8 T	-10.14	6.8	-0.02	0.0012	0.0017	0	0.435		no
II-1	-12.7	7.2	0	4	-0.0006	-0.78	0.15	0	yes
II-2	-12.7	7.2	0	4	-0.0006	-0.78	0.15	0.15	yes
III-1	-12.1	7.2	0	1.8	-0.0019	-0.39	0.75	0.47	no
III-2	-11.5	9.7	-0.08	1.3	-0.0015	-0.39	1.15	0.63	no
III-3	-11.2	7	-0.04	1.3	-0.0015	-0.69	1.15	0.79	no
III-4	-11.2	7	-0.01	1.7	-0.0014	-0.69	0.85	0.94	no
III-5	-10.5	7.7	0	2.93	-0.0005	-0.69	0.33	1.10	no
III-6	-10.5	9.7	0.05	1.7	-0.0008	-0.87	0.86	1.26	no
IV	11.5	6.8	0	4.79	0	-0.4	0.24		yes
V	6	9	0	1.5	-0.004	-0.37	0.99		yes
VI-1	-9.04	8.3	0	0.78	0.0019	-1.16	2.24	0	no
VI-2	-9.04	8.3	0	0.78	0.0013	-0.76	2.24	0.10	no
VII	-11	7	0	3.63	-0.0102	0	0.28		yes
VIII	-11.5	7	0	10.57	0.005	-2.7	0.17		yes
IX-1	-11.4	15.4	0	19.2	0	-0.58	-0.24	0	no
IX-2	-11.1	15.4	0	20	0	-0.73	-0.26	0.09	no
IX-3	-10.9	15.4	0	21.5	0	-0.73	-0.24	0.19	no
IX-4	-10.9	15.4	0	21.5	0	-0.73	-0.24	0.29	no
IX-5	-10.6	15.4	0	21.6	0	-0.73	-0.24	0.39	no
IX-6	-10.6	15.4	0	21.6	0	-0.73	-0.24	0.49	no
IX-7	-10.6	15.4	0	21.6	0	-0.73	-0.24	0.58	no
X-1	-8.3	10	0	0.003	0.002	-1.53	3.8	0	yes
X-2	-8.6	10	0	0.003	0.002	-1.53	3.7	0.09	yes
XI-1	-7.05	11.5	-0.03	0.039	0	-0.47	0.112	0	no
XI-2	-6.94	9.93	0	0.035	0	-0.3	0.2	0.08	no
XII	-9.7	7	0	0.099	0	0.08	0.036		no
XIII-1	-12	9.9	-0.055	0.028	0.0012	-2.16	2.06	0	no
XIII-2	-12	9.9	-0.055	0.028	0.0012	-2.16	2.12	0.12	no
XIII-3	-11.5	9.9	-0.045	0.028	0.0012	-2.12	2.15	0.22	no
XIII-4	-12	9.9	-0.045	0.028	0.0012	-2.1	2.16	0.22	no
XIII-5	-12	9.9	-0.045	0.028	0.0012	-2.1	2.16	0.22	no
XIII-6	-12	9.9	-0.045	0.028	0.0012	-2.1	2.16	0.22	no
XIV	-14.2	10.7	0.008	0.011	-0.0004	-0.34	0.095		no

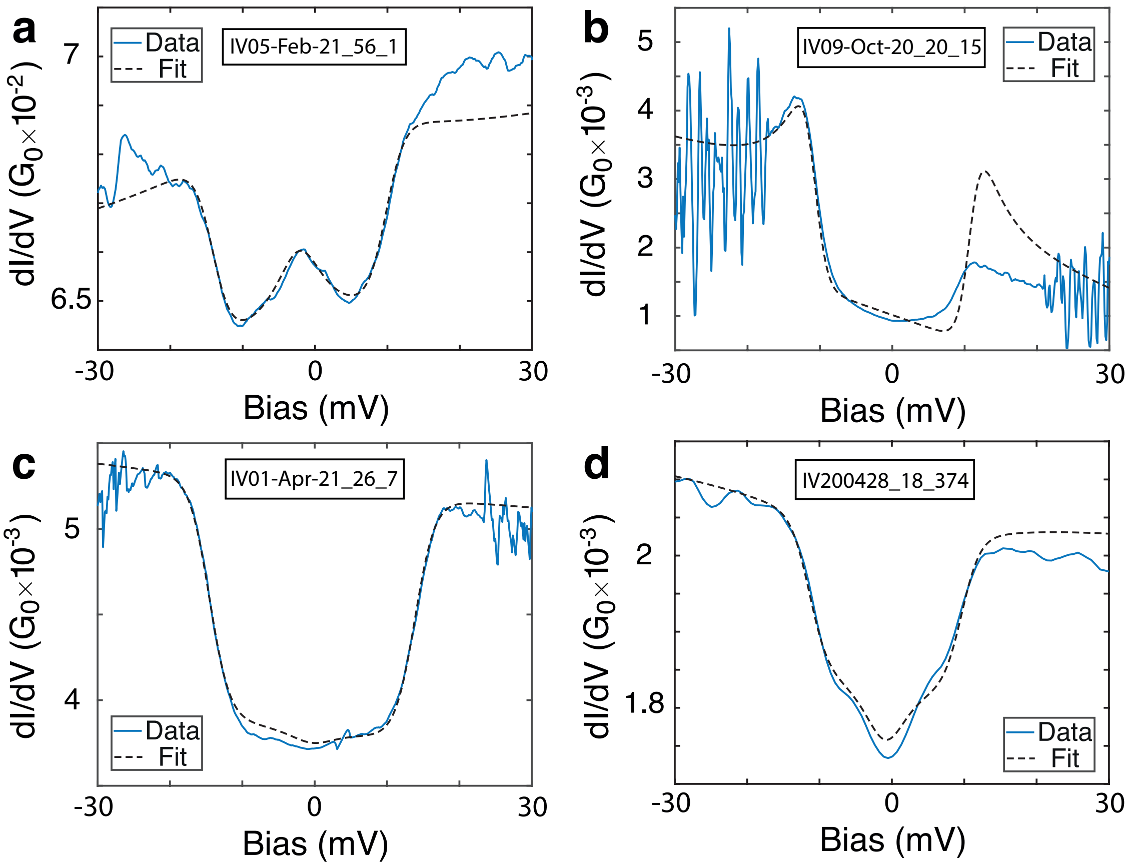

Figure S8: Four dI/dV spectra from different breaking traces. a) dI/dV from series XIII-1 with zero-bias contributions ascribed to a triplet ground state. b) dI/dV from series VII with large bias overshoots at the steps positions indicating a singlet ground state. c) dI/dV from series XIV can be both fitted with a singlet and a triplet ground state. d) dI/dV from series III-6 with another step opening around zero-bias with stretching; this feature can be reproduced in the model using a positive Kondo contribution J_{ρ_s}.

For fitting, we set the program to use both 3rd order and enable interactions of electrons which originate and end in the same electrode (checkbox called 3rd order scattering with other spin systems in the program). Most spectra can be fitted using a negative exchange coupling (ferromagnetic coupling/triplet ground state), and most are better fitted without including the rates equations in the model. Thus, the two spectra with a positive exchange coupling (anti-ferromagnetic coupling/singlet ground state) are described by the model with large bias overshoots (shown in Fig. 1c of the main text and Fig. S8b that are better fitted including the rates equations. Another indication for triplet ground state spectra is the zero-bias conductance enhancement (Fig. 4b of the main text and Fig. S8a that can be captured

by the model using a ferromagnetic exchange coupling.

We fix the value of the g-factor to be 2 in all of the fits as a different value in the carbon lattice of PAH is unlikely. The value $g = 2$ is the best fit to capture the evolution of the steps in the dI/dV spectra around 10 meV at different magnetic fields (IV with series number I, shown in Fig. 4b of the main text) but the opening of the zero-bias dip is better reproduced with a value larger than 2. In the Ternes model, the g-factor is the only parameter that has an effect on the width and height of the zero-bias dip (in the case of ferromagnetic couplings between the spins) but its origin may not be captured in the perturbative approach of the model. Most of the temperatures deduced from the model are consistent with our estimation of 7 K, but some of them are higher (IV with series number IX-1 with 15 K for example). The temperature during the measurement of a single breaking trace is stable.

The exchange coupling, J_{ex}, is almost constant across a breaking series as can be seen in Table S1. In the series III-1 to 6, J_{ex} decreases from -12.1 mV (with a large Kondo contribution around zero bias) to -10.5 mV (without this Kondo contributions). This is also seen in the IV's from breaking series IX-1 to 7 where the exchange coupling starts at -11.4 mV to end with a value of -10.6 mV.

The slope of the background conductance is captured by the b parameter, introducing an asymmetry over the bias voltage range. This asymmetry is also present in the high bias-range, beyond the IETS steps. Inside the steps, the slope is different and no parameters in the model allow to reproduce this feature. The same applies to asymmetries of the zero-bias features. In particular, the bias overshoots present an asymmetry with different amplitudes for the overshoots (see Fig. S8b).

We observe the opening of a step around zero bias in the spectra of Fig. S8d with mechanical stretching reminding the effect of Fig. 4b of the main text without magnetic field applied. This step can be reproduced in the Ternes model using a positive Kondo scattering term J_{ρ_s}. The same is used for the IV from breaking series XIV in Fig. S8c.

c. 1-OS monoradical

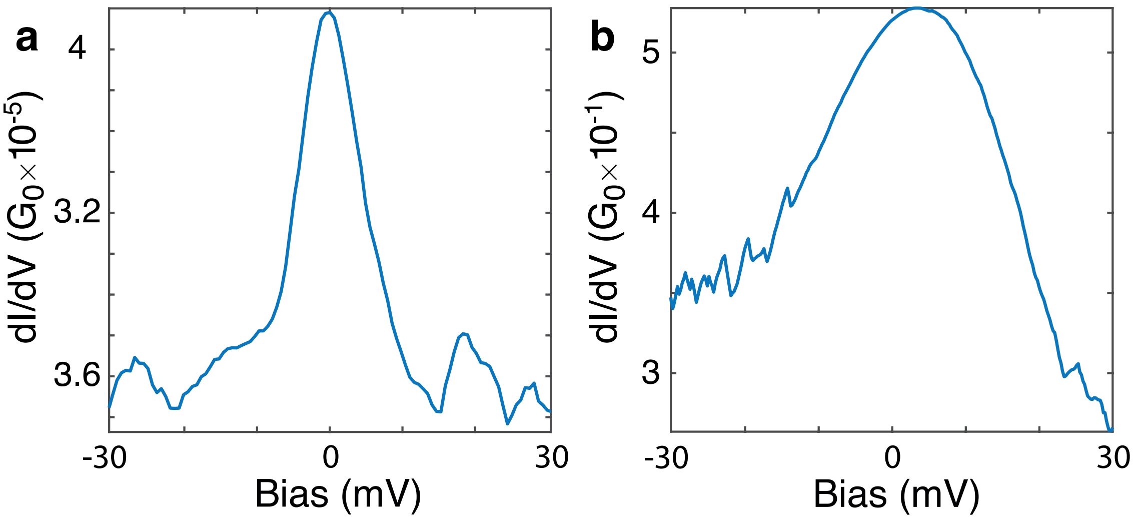

Figure S9: Two zero-bias resonances in the dI/dV spectra for two different breaking traces with an 1-OS molecule. The resonance on the left has an FWHM of 7 mV corresponding to a Kondo temperature of 34 K, while the peak on the right is more asymmetric and has a width of 23 mV (T_{K} = 131 K). The left spectrum is compatible with Kondo physics while the right one most likely does not originate from the same physics (see discussion in the text).

In the 1-OS molecular junction we also observed zero-bias peaks in the dI/dV spectra measured. A typical resonance is shown in Fig. S9a with a FWHM in the same energy range as features observed in 2-OS molecular junctions (around 7 meV). However, the range of the FWHM extracted from the fits of these spectra is larger than that displayed in Fig. 3b of the main text. An example is shown in Fig. S9b where the resonance is found to have an FWHM of 23 meV corresponding to Kondo temperatures of hundreds of K. Most likely, these signals do not arise from Kondo physics and are thereby excluded from the main text figure.

We also observe that mechanical manipulation of the zero-bias peak can shift the center of the resonance with electrodes spacing (see S10); as a consequence the center is no longer found at $V = 0$ (see Fig. S9b and S10). This behaviour is different from the observations for the 2-OS molecule where the width and height of the peak reduces while the center of the resonance

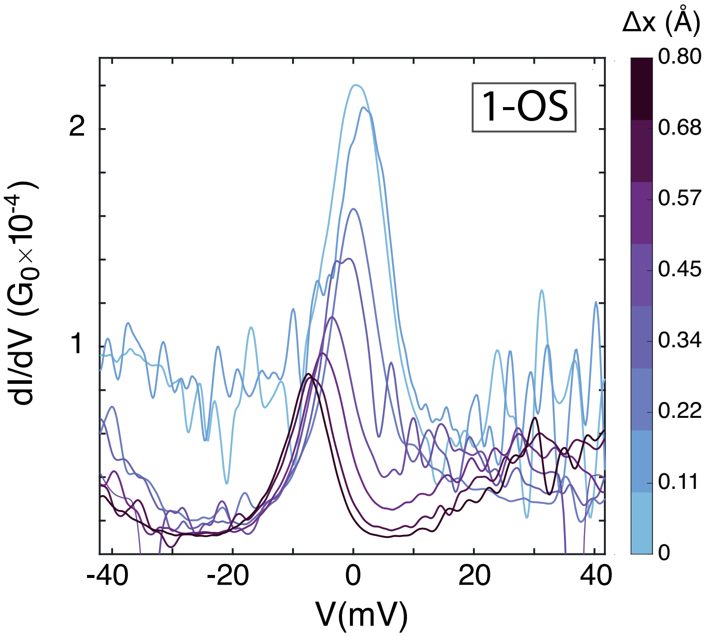

Figure S10: Breaking series from 1-OS reference measurement displaying a zero-bias resonance persisting along with the stretching of the electrodes. The center of the resonance is shifting from 0 mV on the first IV of the series to -8 mV after breaking the electrodes 0.8 Å apart. This result is different from the comportment observed for the 2-OS Kondo feature displayed in Fig. 3d of the main text.

stay close to zero-bias (as shown in Fig. 3d of main text). Another interesting feature of the 1-OS measurements is the presence of the large peaks in the spectra, symmetrically located around zero bias as shown in Fig. S11a. In some spectra, a small zero-bias resonance between these large structure was present (see Fig. S11b) which could indicate the presence of a Kondo resonance.

The features in the aforementioned paragraph can be explained by the proximity of an energy level close to the Fermi energy. When this energy level approaches the Fermi level of the electrodes, enhancement of the conductance through the molecule appears in the IV's. This energy level alignment depends on the injection point of charge carriers in the molecule and on the conformation in the junction; consequently, not all IV's exhibit this behavior. It would be interesting to understand which configurations lead to this better level alignment. Future work and calculations are needed to address this crucial aspect in single-molecule electronics.

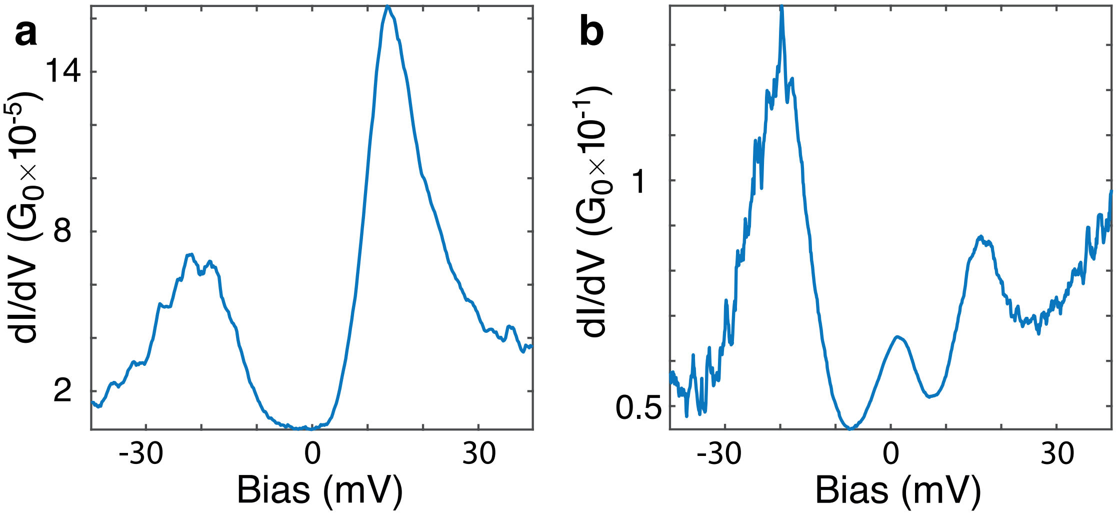

Figure S11: Two dI/dV spectra with resonant structures, symmetric with respect to zero-bias at different positions of the same breaking traces of 1-OS molecular junctions. The right spectrum also shows a zero-bias resonance which may be an indication that the 1-OS molecule has an odd number of electrons ($S = 1/2$ Kondo).

References

(1) Zeng, Z. et al. Stable Tetrabenzo-Chichibabin's Hydrocarbons: Tunable Ground State and Unusual Transition between Their Closed-Shell and Open-Shell Resonance Forms. *Journal of the American Chemical Society* **2012**, *134*, 14513–14525, PMID: 22889277.

(2) Lee, H.; Jo, M.; Yang, G.; Jung, H.; Kang, S.; Park, J. Highly efficient dual anthracene core derivatives through optimizing side groups for blue emission. *Dyes and Pigments* **2017**, *146*, 27–36.

(3) Tanaka, K.; Kishigami, S.; Toda, F. A new method for coupling aromatic aldehydes and ketones to produce. alpha.-glycols using zinc-zinc dichloride in aqueous solution and in the solid state. *The Journal of Organic Chemistry* **1990**, *55*, 2981–2983.

(4) Ternes, M. Spin excitations and correlations in scanning tunneling spectroscopy. *New Journal of Physics* **2015**, *17*, 063016.
